# 2-(1*H*-Tetra­zol-5-yl)benzonitrile

**DOI:** 10.1107/S1600536808008027

**Published:** 2008-04-10

**Authors:** Zheng Xing, Guang-Fan Han, Wei-Feng Zhu, Yu-Yuan Zhao

**Affiliations:** a School of Materials Science and Engineering, Jiangsu University of Science, and Technology, Zhenjiang, Jiangsu 212003, People’s Republic of China

## Abstract

The title compound, C_8_H_5_N_5_, was synthesized from phthalonitrile. The benzonitrile and tetra­zole rings are inclined at a dihedral angle of 37.14 (11)°. In the crystal structure, inter­molecular N—H⋯N hydrogen bonds link the tetra­zole rings of adjacent mol­ecules, forming chains along the *a* axis.

## Related literature

For backgound to the chemisty of tetra­zoles, see: Bekhit *et al.* (2004 ▶); Aykut İkizler & Sancak (1992 ▶, 1995 ▶, 1998 ▶); Rajasekaran & Thampi (2004 ▶); Satyanarayana *et al.* (2006 ▶); Schmidt & Schieffer (2003 ▶); Upadhayaya *et al.* (2004 ▶); Wexler *et al.* (1996 ▶).
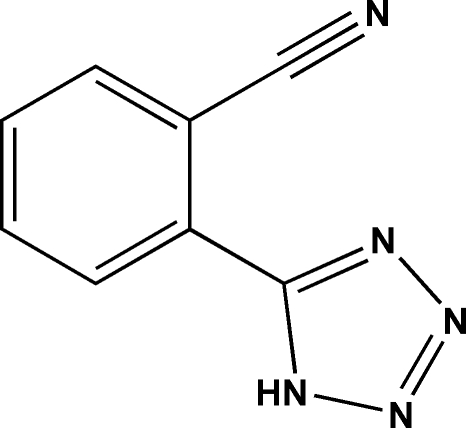


## Experimental

### 

#### Crystal data

C_8_H_5_N_5_
*M* *_r_* = 171.17
Monoclinic, 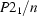

*a* = 4.9281 (10) Å
*b* = 6.5420 (13) Å
*c* = 24.867 (5) Å
β = 95.27 (3)°
*V* = 798.3 (3) Å^3^
*Z* = 4
Mo *K*α radiation
μ = 0.10 mm^−1^
*T* = 293 (2) K
0.25 × 0.07 × 0.07 mm

#### Data collection

Rigaku SCXmini diffractometer
Absorption correction: multi-scan (*CrystalClear*; Rigaku, 2005 ▶) *T*_min_ = 0.993, *T*_max_ = 0.996
6844 measured reflections
1553 independent reflections
1167 reflections with *I* > 2σ(*I*)
*R*_int_ = 0.061

#### Refinement

*R*[*F* ^2^ > 2σ(*F* ^2^)] = 0.055
*wR*(*F* ^2^) = 0.138
*S* = 1.10
1553 reflections
122 parameters
H atoms treated by a mixture of independent and constrained refinement
Δρ_max_ = 0.21 e Å^−3^
Δρ_min_ = −0.24 e Å^−3^

### 

Data collection: *CrystalClear* (Rigaku, 2005 ▶); cell refinement: *CrystalClear*; data reduction: *CrystalClear*; program(s) used to solve structure: *SHELXS97* (Sheldrick, 2008 ▶); program(s) used to refine structure: *SHELXL97* (Sheldrick, 2008 ▶); molecular graphics: *SHELXTL* (Sheldrick, 2008 ▶); software used to prepare material for publication: *SHELXTL*.

## Supplementary Material

Crystal structure: contains datablocks I, New_Global_Publ_Block. DOI: 10.1107/S1600536808008027/sj2471sup1.cif

Structure factors: contains datablocks I. DOI: 10.1107/S1600536808008027/sj2471Isup2.hkl

Additional supplementary materials:  crystallographic information; 3D view; checkCIF report

## Figures and Tables

**Table 1:** Hydrogen-bond geometry (Å, °)

*D*—H⋯*A*	*D*—H	H⋯*A*	*D*⋯*A*	*D*—H⋯*A*
N2—H2⋯N5^i^	0.92 (3)	1.91 (3)	2.820 (2)	172 (2)
N2—H2⋯N4^i^	0.92 (3)	2.60 (3)	3.374 (2)	142 (2)

Symmetry code: (i) 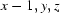.

**Fig. 1.**
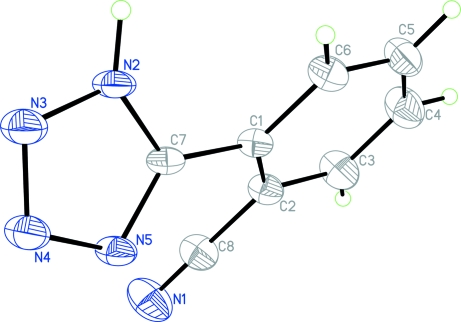
A view of the title compound with the atomic numbering scheme. Displacement ellipsoids are drawn at the 30% probability level.

**Fig. 2.**
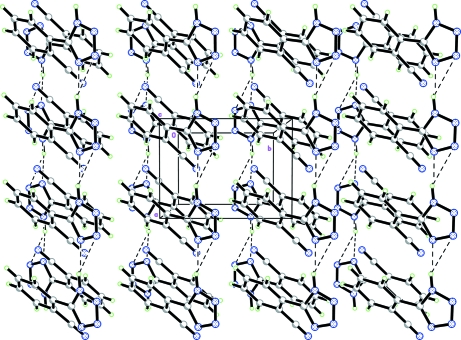
The crystal packing of the title compound viewed along the c axis. Hydrogen bonds are drawn as dashed lines.

## supplementary crystallographic information

### Comment

Nitriles are close relatives of azoles and hydrazones and are parent compounds for the preparation of various functional organic materials having triazole, imidazole or tetrazole rings (Aykut İkizler & Sancak, 1992, 1995, 1998). Tetrazoles find wide application in the synthesis of medicinal products such as antihypertensive agents (Wexler *et al.*, 1996; Schmidt & Schieffer, 2003; Satyanarayana *et al.*, 2006), resolvents (Bekhit *et al.*, 2004), anaesthetics (Rajasekaran & Thampi, 2004) and antifungal agents (Upadhayaya *et al.*, 2004). We report herein the crystal structure of the title compound, 2-(1*H*-tetrazol-5-yl)benzonitrile, Fig 1 with its crystal packing shown in Figure 2, Table 1.

### Experimental

Phthalonitrile (1.28 g, 0.01 mol), sodium azide (0.975 g, 0.015 mol), ammonium chloride (0.605 g, 0.011 mol) and DMF (15 ml) were added in a flask and reacted at 120 °C with stirring for 24 h. A mass of white solid was collected after solvents removed. The crude product was recrystallized by slowly evaporating a mixed solution of ethanol and water (2:1) to yield colorless prism-like crystals, suitable for X-ray analysis.

### Refinement

The H2 atom bound to N2 was located in a difference map and was refined freely. Other H atoms were placed in calculated positions, with C—H = 0.93 Å and included in the final cycles of refinement using a riding model, with *U*_iso_(H) = 1.2Ueq

### Crystal data

**Table:**

C_8_H_5_N_5_	*F*_000_ = 352
*M**_r_* = 171.17	*D*_x_ = 1.424 Mg m^−^^3^
Monoclinic, *P*2_1_/*n*	Mo *K*α radiation λ = 0.71073 Å
Hall symbol: -P 2yn	Cell parameters from 6584 reflections
*a* = 4.9281 (10) Å	θ = 3.1–28.8º
*b* = 6.5420 (13) Å	µ = 0.10 mm^−^^1^
*c* = 24.867 (5) Å	*T* = 293 (2) K
β = 95.27 (3)º	Prism, colorless
*V* = 798.3 (3) Å^3^	0.25 × 0.07 × 0.07 mm
*Z* = 4	

### Data collection

**Table:**

Rigaku SCXmini diffractometer	1553 independent reflections
Radiation source: fine-focus sealed tube	1167 reflections with *I* > 2σ(*I*)
Monochromator: graphite	*R*_int_ = 0.061
Detector resolution: 13.6612 pixels mm^-1^	θ_max_ = 26.0º
*T* = 293(2) K	θ_min_ = 3.2º
ω scans	*h* = −6→6
Absorption correction: multi-scan(CrystalClear; Rigaku, 2005)	*k* = −8→8
*T*_min_ = 0.993, *T*_max_ = 0.996	*l* = −30→30
6844 measured reflections	

### Refinement

**Table:**

Refinement on *F*^2^	Hydrogen site location: inferred from neighbouring sites
Least-squares matrix: full	H atoms treated by a mixture of independent and constrained refinement
*R*[*F*^2^ > 2σ(*F*^2^)] = 0.055	*w* = 1/[σ^2^(*F*_o_^2^) + (0.0619*P*)^2^ + 0.1285*P*] where *P* = (*F*_o_^2^ + 2*F*_c_^2^)/3
*wR*(*F*^2^) = 0.138	(Δ/σ)_max_ < 0.001
*S* = 1.10	Δρ_max_ = 0.21 e Å^−^^3^
1553 reflections	Δρ_min_ = −0.24 e Å^−^^3^
122 parameters	Extinction correction: SHELXL
Primary atom site location: structure-invariant direct methods	Extinction coefficient: 0.044 (14)
Secondary atom site location: difference Fourier map	

### Special details

**Table:**

Geometry. All e.s.d.'s (except the e.s.d. in the dihedral angle between two l.s. planes) are estimated using the full covariance matrix. The cell e.s.d.'s are taken into account individually in the estimation of e.s.d.'s in distances, angles and torsion angles; correlations between e.s.d.'s in cell parameters are only used when they are defined by crystal symmetry. An approximate (isotropic) treatment of cell e.s.d.'s is used for estimating e.s.d.'s involving l.s. planes.
Refinement. Refinement of *F*^2^ against ALL reflections. The weighted *R*-factor *wR* and goodness of fit *S* are based on *F*^2^, conventional *R*-factors *R* are based on *F*, with *F* set to zero for negative *F*^2^. The threshold expression of *F*^2^ > σ(*F*^2^) is used only for calculating *R*-factors(gt) *etc*. and is not relevant to the choice of reflections for refinement. *R*-factors based on *F*^2^ are statistically about twice as large as those based on *F*, and *R*- factors based on ALL data will be even larger.

### Fractional atomic coordinates and isotropic or equivalent isotropic displacement parameters (Å^2^)

**Table:**

	*x*	*y*	*z*	*U*_iso_*/*U*_eq_	
C1	0.5249 (4)	0.5054 (3)	0.13602 (8)	0.0345 (5)	
C2	0.6569 (4)	0.5277 (3)	0.08892 (8)	0.0403 (5)	
C3	0.6021 (5)	0.6960 (4)	0.05536 (10)	0.0548 (7)	
H3	0.6925	0.7116	0.0244	0.066*	
C4	0.4141 (5)	0.8390 (4)	0.06821 (11)	0.0608 (7)	
H4	0.3762	0.9509	0.0457	0.073*	
C5	0.2821 (5)	0.8174 (4)	0.11410 (11)	0.0539 (6)	
H5	0.1537	0.9140	0.1223	0.065*	
C6	0.3384 (4)	0.6532 (3)	0.14819 (9)	0.0441 (6)	
H6	0.2504	0.6416	0.1796	0.053*	
C7	0.5736 (4)	0.3299 (3)	0.17202 (8)	0.0323 (5)	
C8	0.8482 (5)	0.3773 (4)	0.07280 (9)	0.0476 (6)	
N1	0.9990 (5)	0.2612 (4)	0.05847 (9)	0.0701 (7)	
N2	0.3814 (3)	0.2405 (3)	0.19806 (7)	0.0399 (5)	
N3	0.4920 (3)	0.0893 (3)	0.22916 (8)	0.0480 (5)	
N4	0.7498 (3)	0.0881 (3)	0.22217 (7)	0.0458 (5)	
N5	0.8068 (3)	0.2363 (3)	0.18650 (7)	0.0380 (5)	
H2	0.195 (6)	0.253 (4)	0.1939 (10)	0.072 (8)*	

### Atomic displacement parameters (Å^2^)

**Table:**

	*U*^11^	*U*^22^	*U*^33^	*U*^12^	*U*^13^	*U*^23^
C1	0.0263 (10)	0.0405 (11)	0.0363 (11)	0.0019 (9)	0.0016 (8)	0.0010 (9)
C2	0.0345 (12)	0.0463 (12)	0.0407 (12)	0.0040 (10)	0.0062 (9)	0.0018 (10)
C3	0.0598 (16)	0.0595 (15)	0.0473 (14)	0.0110 (12)	0.0171 (12)	0.0137 (12)
C4	0.0658 (17)	0.0558 (15)	0.0611 (17)	0.0139 (13)	0.0072 (14)	0.0187 (13)
C5	0.0491 (14)	0.0480 (14)	0.0642 (16)	0.0144 (11)	0.0041 (12)	−0.0003 (12)
C6	0.0397 (12)	0.0507 (13)	0.0425 (13)	0.0070 (10)	0.0069 (10)	−0.0014 (10)
C7	0.0221 (9)	0.0434 (11)	0.0319 (11)	0.0004 (8)	0.0055 (8)	−0.0021 (9)
C8	0.0476 (13)	0.0573 (15)	0.0395 (13)	0.0069 (12)	0.0127 (10)	0.0069 (11)
N1	0.0705 (15)	0.0809 (15)	0.0623 (15)	0.0288 (13)	0.0242 (12)	0.0062 (12)
N2	0.0203 (9)	0.0531 (11)	0.0468 (11)	0.0024 (8)	0.0058 (8)	0.0107 (8)
N3	0.0306 (9)	0.0600 (12)	0.0540 (12)	0.0029 (9)	0.0074 (8)	0.0166 (10)
N4	0.0296 (9)	0.0583 (12)	0.0494 (11)	0.0047 (8)	0.0042 (8)	0.0139 (9)
N5	0.0238 (8)	0.0510 (10)	0.0400 (10)	0.0035 (8)	0.0065 (7)	0.0070 (8)

### Geometric parameters (Å, °)

**Table:**

C1—C6	1.386 (3)	C5—H5	0.9300
C1—C2	1.399 (3)	C6—H6	0.9300
C1—C7	1.462 (3)	C7—N5	1.323 (2)
C2—C3	1.393 (3)	C7—N2	1.331 (2)
C2—C8	1.445 (3)	C8—N1	1.142 (3)
C3—C4	1.375 (3)	N2—N3	1.340 (2)
C3—H3	0.9300	N2—H2	0.92 (3)
C4—C5	1.372 (3)	N3—N4	1.298 (2)
C4—H4	0.9300	N4—N5	1.361 (2)
C5—C6	1.381 (3)		
			
C6—C1—C2	118.61 (19)	C6—C5—H5	119.8
C6—C1—C7	119.24 (18)	C5—C6—C1	120.5 (2)
C2—C1—C7	122.13 (17)	C5—C6—H6	119.7
C3—C2—C1	120.31 (19)	C1—C6—H6	119.7
C3—C2—C8	117.86 (19)	N5—C7—N2	107.65 (17)
C1—C2—C8	121.81 (19)	N5—C7—C1	128.31 (17)
C4—C3—C2	119.7 (2)	N2—C7—C1	123.99 (17)
C4—C3—H3	120.1	N1—C8—C2	177.8 (2)
C2—C3—H3	120.1	C7—N2—N3	109.61 (16)
C5—C4—C3	120.3 (2)	C7—N2—H2	130.9 (16)
C5—C4—H4	119.9	N3—N2—H2	118.7 (16)
C3—C4—H4	119.9	N4—N3—N2	106.16 (16)
C4—C5—C6	120.5 (2)	N3—N4—N5	110.25 (16)
C4—C5—H5	119.8	C7—N5—N4	106.33 (15)
			
C6—C1—C2—C3	0.4 (3)	C6—C1—C7—N5	−141.7 (2)
C7—C1—C2—C3	179.22 (19)	C2—C1—C7—N5	39.5 (3)
C6—C1—C2—C8	−178.3 (2)	C6—C1—C7—N2	35.6 (3)
C7—C1—C2—C8	0.5 (3)	C2—C1—C7—N2	−143.2 (2)
C1—C2—C3—C4	−1.1 (4)	N5—C7—N2—N3	−0.1 (2)
C8—C2—C3—C4	177.7 (2)	C1—C7—N2—N3	−177.85 (18)
C2—C3—C4—C5	0.5 (4)	C7—N2—N3—N4	0.3 (2)
C3—C4—C5—C6	0.7 (4)	N2—N3—N4—N5	−0.4 (2)
C4—C5—C6—C1	−1.3 (4)	N2—C7—N5—N4	−0.2 (2)
C2—C1—C6—C5	0.8 (3)	C1—C7—N5—N4	177.48 (19)
C7—C1—C6—C5	−178.05 (19)	N3—N4—N5—C7	0.4 (2)

### Hydrogen-bond geometry (Å, °)

**Table:**

*D*—H···*A*	*D*—H	H···*A*	*D*···*A*	*D*—H···*A*
N2—H2···N5^i^	0.92 (3)	1.91 (3)	2.820 (2)	172 (2)
N2—H2···N4^i^	0.92 (3)	2.60 (3)	3.374 (2)	142 (2)

Symmetry codes: (i) *x*−1, *y*, *z*.
